# Development of a nomogram model based on spleen volume change to predict high-risk esophageal varices in patients with liver cirrhosis

**DOI:** 10.3389/fsurg.2025.1699002

**Published:** 2026-01-13

**Authors:** Zuo-Jun Li, Jing Chen, Li Li, Yu-Tao Zhan

**Affiliations:** 1 Department of Infectious Disease, Beijing Tongren Hospital, Capital Medical University, Beijing, China; 2 Department of Gastroenterology, Beijing Tongren Hospital, Capital Medical University, Beijing, China

**Keywords:** esophageal varices, liver cirrhosis, nomogram, risk, spleen volume

## Abstract

**Background:**

Esophageal variceal (EV) rupture is a life-threatening complication of liver cirrhosis. Although upper gastrointestinal endoscopy is recommended for routine screening and risk assessment of EV bleeding, it is an invasive and often unpleasant procedure. This study aims to develop a non-invasive nomogram model based on spleen volume changes to predict the presence of high-risk esophageal varices (HREVs).

**Methods:**

A total of 150 patients with liver cirrhosis (mean age 62.3 ± 10.0 years; 95 men and 55 women) who underwent upper gastrointestinal endoscopy were retrospectively included. Spleen volume was measured using abdominal computed tomography. Predictors were identified through multivariate logistic regression and subsequently used to construct a nomogram model. The discriminative ability, calibration ability, and clinical utility were assessed. Internal validation was performed using 1,000 bootstrap resampling iterations.

**Results:**

Based on endoscopic findings, 74 patients were categorized into the HREV group and 76 patients were categorized into the non-HREV group. Multivariate regression identified three independent predictors of HREV: the presence of ascites [odds ratio (OR) = 2.656, 95% confidence interval (CI): 1.224–5.763], prothrombin time (OR = 1.217, 95% CI: 1.043–1.419), and spleen volume enlargement rate (OR = 1.589, 95% CI: 1.276–1.979). These variables were incorporated into the nomogram model. The area under the receiver operating characteristic curve of the nomogram model was 0.793 (95% CI: 0.723–0.863), outperforming previously reported models, such as the platelet-to-spleen volume ratio (0.724), platelet-to-spleen diameter ratio (0.673), aspartate aminotransferase-to-platelet ratio index (0.590), and aspartate aminotransferase-to-alanine aminotransferase ratio (0.558). At a probability cutoff of 0.421, the nomogram demonstrated a sensitivity of 0.797, a specificity of 0.671, a positive predictive value of 0.702, a negative predictive value of 0.773, and an accuracy of 0.733. Internal validation yielded a *C*-index of 0.779 (95% CI: 0.714–0.853). Overall, the nomogram model exhibited good calibration and favorable clinical utility.

**Conclusion:**

The nomogram incorporating ascites, prothrombin time, and spleen volume enlargement rate effectively predicts HREVs in patients with liver cirrhosis. This non-invasive and user-friendly tool offers an efficient approach for timely HREV evaluation and preventive treatment of variceal bleeding.

## Introduction

Liver cirrhosis, the end stage of chronic liver diseases, is frequently complicated by portal hypertension and the development of extensive collateral circulation (1). Esophageal varices (EVs) are one of the most common manifestations of portal hypertension. Acute bleeding from ruptured EVs represents a critical and life-threatening complication in patients with liver cirrhosis, with an annual incidence of 5%–15% and a mortality rate of 15%–25% (2, 3). According to the Baveno criteria, patients with high-risk EVs (HREVs) are at an increased risk of variceal rupture and should be treated with non-selective beta blockers (NSBBs) and/or endoscopic band ligation to prevent bleeding (4, 5). Therefore, timely detection and bleeding risk stratification of EVs are crucial for preventive treatment and improving prognosis in patients with liver cirrhosis.

Upper gastrointestinal endoscopy remains the gold standard for diagnosing EVs and is recommended for routine screening in patients with liver cirrhosis (6). Risk stratification of HREVs primarily depends on endoscopic findings, such as the size of the varices and the presence of red color signs (3). However, endoscopy is an invasive and costly procedure associated with procedural risks, patient discomfort, and psychological burden, which may reduce patient compliance (7). Consequently, many patients do not undergo endoscopic evaluation until the disease progresses to HREVs or the occurrence of variceal bleeding. Thus, the development of a non-invasive model for accurately predicting HREVs is of significant clinical importance for early screening and assessing bleeding risk.

In patients with liver cirrhosis, spleen size is typically enlarged due to collateral circulation and is associated with the presence of portal hypertension and EVs (8, 9). Spleen volume (SV), as measured by computed tomography (CT) or magnetic resonance imaging (MRI), is significantly greater in patients with EVs than in those without EVs (10) and is further increased in patients at high risk of variceal bleeding compared to those at lower risk (11, 12). Therefore, SV has been explored as a potential predictor of EVs or HREVs in patients with liver cirrhosis. The ratio of SV to platelet count (or platelet-to-SV ratio) has shown good predictive performance for EVs or HREVs (10, 13, 14). Furthermore, when liver and spleen volume measurements are combined, the ratio of SV to right liver lobe volume has shown superior predictive performance for variceal bleeding compared with other parameters (12). However, previous studies have used composite indices that combine spleen volume with additional variables to predict HREVs. Given the characteristic spleen enlargement in patients with liver cirrhosis, we hypothesized that the spleen volume enlargement rate (SVER), calculated from actual and standard spleen volumes, could serve as a novel predictor of bleeding risk in EVs. Despite this potential, the predictive value of the SVER has been insufficiently studied. The present study aims to develop a non-invasive nomogram model based on spleen volume changes and laboratory data to predict HREVs in patients with liver cirrhosis.

## Materials and methods

### Participants

This study retrospectively analyzed patients diagnosed with liver cirrhosis who underwent upper gastrointestinal endoscopy and upper abdominal computed tomography (CT) at Beijing Tongren Hospital, Capital Medical University, between June 2010 and December 2023. The inclusion criteria were as follows: (1) age 18 years or older; (2) diagnosis of liver cirrhosis based on clinical symptoms, laboratory tests, and imaging examinations according to the Chinese guidelines on the management of liver cirrhosis (15), as none of the patients underwent liver biopsy; and (3) availability of complete endoscopy, CT, and laboratory examination data. The exclusion criteria were as follows: (1) suspected liver tumors; (2) history of esophageal bleeding or endoscopic or surgical treatment; (3) history of hepatectomy or splenectomy; (4) known hematological disorders affecting spleen size; (5) other benign conditions that may alter spleen volume, such as hemolytic anemias, hemoglobinopathies, splenic artery embolism, and portal vein thrombosis; (6) severe cardiovascular disease; and (7) significant weight loss or malnutrition. This study was conducted in accordance with the Declaration of Helsinki and approved by the Ethics Committee of Beijing Tongren Hospital (TRE02024-KY066). The requirement for informed consent was waived due to the retrospective nature of this study.

### Collection of basic information and clinical laboratory data

Baseline information was collected, including sex, age, body weight (BW), body height (BH), Child–Pugh class, presence of ascites, and etiology of liver cirrhosis. Body surface area (BSA) was calculated using the Mosteller formula: BSA = [BW (kg) × BH (cm)/3,600]^1/2^ (16). Clinical laboratory tests included routine blood tests, coagulation function assessments, and evaluations of liver and kidney function. The collected laboratory data included white blood cell (WBC) count, red blood cell (RBC) count, platelet count (PLT), hemoglobin, nitrogen, creatinine, albumin, total bilirubin (TBL), alanine aminotransferase (ALT), aspartate aminotransferase (AST), γ-glutamyl transpeptidase (GGT), serum sodium (SNA), prothrombin time (PT), prothrombin activity (PTA), and the prothrombin international normalized ratio (INR). The Model for End-Stage Liver Disease (MELD) score was calculated using the following formula: MELD = 9.57 × ln (creatinine) + 3.78 × ln (bilirubin) + 11.20 × ln (INR) + 6.43, and the final score was rounded to the nearest whole number (17). The platelet-to-spleen volume ratio (PSVR), platelet-to-spleen diameter ratio (PSDR), AST-to-platelet ratio index (APRI), and AST-to-ALT ratio (AAR) were calculated as follows: PSVR = PLT (n/mm^3^)/spleen volume (cm^3^); PSDR = PLT (n/mm^3^)/spleen long diameter (mm); APRI = AST (U/L)/[AST (normal upper limit) × 100]/PLT (10^9^/L); and AAR = AST/ALT (18).

### Definition of HREVs

EVs were classified according to endoscopic findings: grade 1 (straight, small-caliber varices), grade 2 (tortuous veins with a bead-like appearance), and grade 3 (tumor-shaped varices) (19). HREVs were defined according to the Baveno VI standard as grade 2 or grade 3 EVs, grade 1 EVs with red color signs, or grade 1 EVs with Child–Pugh class C cirrhosis (5). Patients without EVs or those with grade 1 EVs that did not meet these criteria were classified as non-HREVs.

### Measurement of spleen volume by CT scanning

Contrast-enhanced upper abdominal CT scanning was conducted using a Brilliance iCT or IQon Spectral CT scanner (Philips Healthcare, Amsterdam, Netherlands) following administration of iopromide (370 mgI/ml; Bayer Healthcare, Berlin, Germany). An experienced radiologist, blinded to the clinical information and laboratory results of the patient, measured the portal vein diameter (PVD), splenic vein diameter (SVD), spleen long diameter (SLD), actual liver volume (CTLV), and actual spleen volume (CTSV). Measurements were performed on portal venous phase images using the Philips IntelliSpace Portal (ISP) workstation. Following initial automated segmentation of the entire liver and spleen by the ISP software, organ boundaries were verified and easily manipulated when necessary using adjustable digital brush or eraser tools to add or subtract tissue volumes, respectively. At this point, the total liver volume and spleen volume were recorded. The SLD was defined as the distance from the superior pole to the inferior line of the spleen on the plane demonstrating the maximum surface area. The spleen volume enlargement rate (SVER) was calculated as follows: SVER = (CTSV−SLV)/SLV, where the standard spleen volume (SLV) was determined as follows: SLV (cm^3^) = 156.8277 × BSA−101.8544 (R^2^ = 0.233).

### Inter-observer reliability

For a subset of 30 randomly selected patients, a second experienced radiologist independently measured the PVD, SVD, SLD, CTLV, and CTSV while blinded to clinical information. Inter-observer reliability of these measurements between the two radiologists was assessed using the interclass correlation coefficient (ICC). In our study, the ICCs for PVD, SVD, SLD, CTLV, and CTSV were 0.93 [95% confidence interval (CI): 0.83–0.97], 0.97 (95% CI: 0.93–0.98), 0.96 (95% CI: 0.92–0.98), 0.91 (95% CI: 0.81–0.95), and 0.94 (95% CI: 0.85–0.97), respectively, indicating excellent inter-observer agreement.

### Statistical analysis

All statistical analyses were performed using STATA 16.0 (StataCorp, TX, USA). Continuous variables were expressed as mean ± standard deviation (SD) and compared using the *t*-test, or as median with interquartile range (IQR) and compared using the Wilcoxon rank-sum test. Categorical variables were compared using the chi-square test. Variables with a *P*-value <0.10 in univariate logistic regression analysis were included in multivariate analysis. Backward stepwise logistic regression was used to identify independent predictors, and a nomogram was constructed to predict HREVs. The discriminative ability of the nomogram model was assessed using receiver operating characteristic curve (ROC) analysis and area under the curve (AUC). Internal validation of the nomogram model was performed using the bootstrap method with 1,000 re-sampling iterations. The optimal cutoff for the nomogram model was determined by maximizing the Youden index, and the corresponding sensitivity, specificity, positive predictive value (PPV), negative predictive value (NPV), and overall accuracy were calculated. The performance of the nomogram model was compared with other non-invasive predictive models, including the PSVR, PSDR, APRI, and AAR, using the DeLong test. The calibration of the nomogram was assessed using a calibration curve and the Hosmer–Lemeshow test. Decision curve analysis (DCA) was performed to evaluate clinical utility. A *P*-value <0.05 was considered statistically significant.

## Results

### Baseline characteristics of all patients

**Figure 1:**
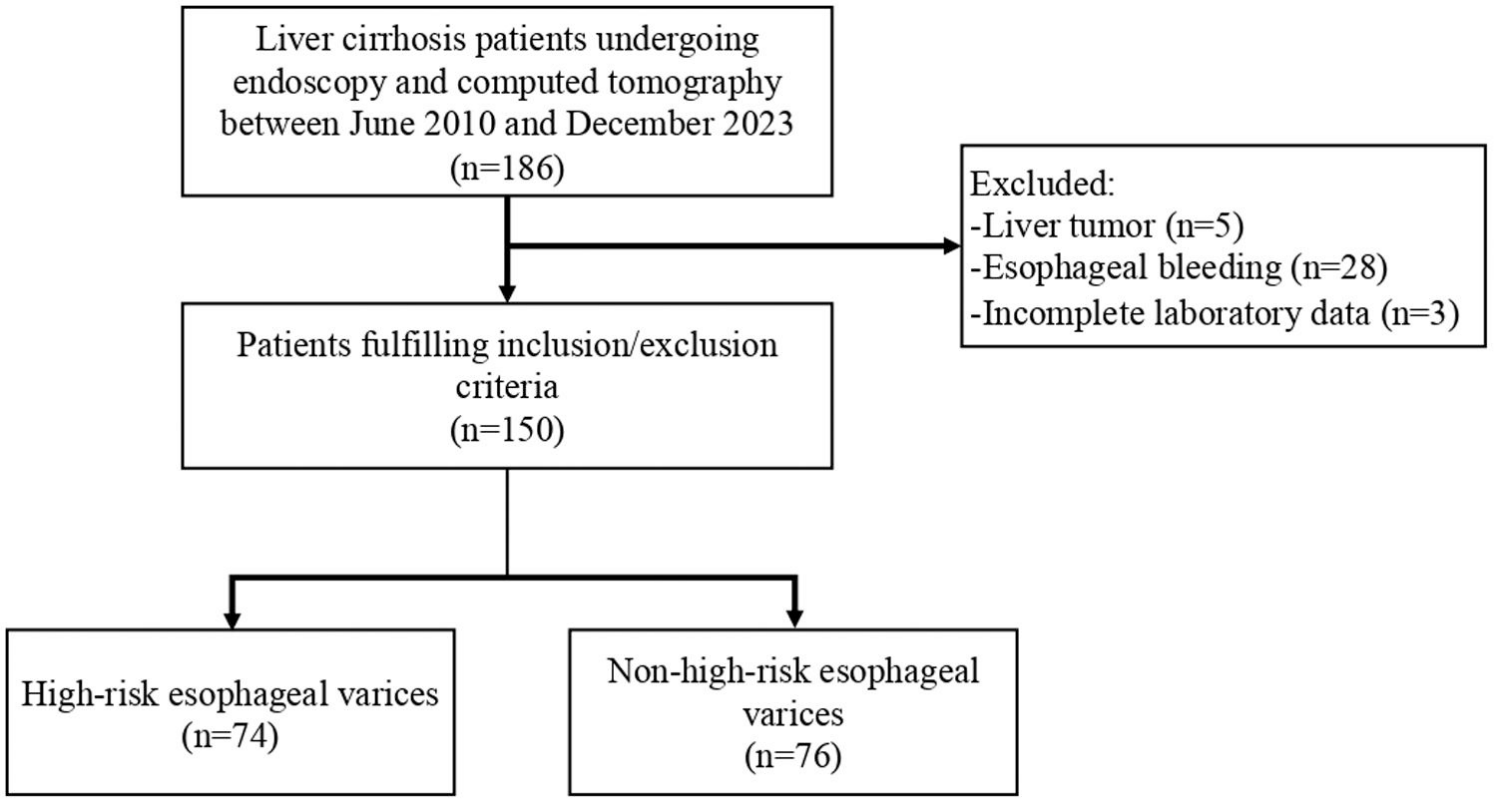
Flow diagram of patient selection.

A total of 150 eligible patients were included in the analysis, of whom 74 were categorized into the HREV group and 76 were classified into the non-HREV group (Figure 1). Among the participants, 95 (63.3%) were men and 55 (36.7%) were women, with a mean age of 62.3 ± 10.0 years. Regarding the severity of liver cirrhosis, 58 (38.7%) patients were classified as Child–Pugh class A, 66 (44.0%) as class B, and 26 (17.3%) as class C. Alcoholic liver disease was the predominant cause of cirrhosis, accounting for 68 (45.3%) cases, followed by primary biliary cirrhosis (21 cases, 14.0%), hepatitis B virus infection (20 cases, 13.3%), cryptogenic cirrhosis (20 cases, 13.3%), autoimmune liver disease (11 cases, 7.3%), non-alcoholic fatty liver disease (5 cases, 3.3%), hepatitis C virus (4 cases, 2.7%), and Budd–Chiari syndrome (1 case, 0.7%). Baseline characteristics of patients in the HREV and non-HREV groups are summarized in Table 1.

**Table 1:** Baseline characteristics of HREV and non-HREV patients.

Variable	Total	HREV	Non-HREV	*P*
Total	150	74	76	
**Sex**				
Male	95 (63.3)	50 (67.8)	45 (59.2)	0.288
Female	55 (36.7)	24 (32.4)	31 (40.8)	
Age (years)	62.3 ± 10.0	61.1 ± 9.6	63.4 ± 10.3	0.150
BMI (kg/m^2^)	22.7 ± 3.8	23.0 ± 3.9	22.3 ± 3.8	0.272
**Ascites**				
Presence	89 (59.3)	55 (74.3)	34 (44.7)	<0.001
Absence	61 (40.7)	19 (25.7)	42 (55.3)	
WBC (10^9^/L)	4.2 (3.2–5.2)	3.9 (3.0–5.2)	4.3 (3.7–5.2)	0.069
RBC (10^12^/L)	3.5 (3.1–3.9)	3.4 (3.0–3.7)	3.5 (3.1–4.0)	0.070
Hemoglobin (g/L)	115 (98–126)	112 (98–125)	119 (98–130)	0.258
PLT (10^9^/L)	104 (70–130)	91 (60–116)	111 (80–142)	0.002
Nitrogen (mmol/L)	4.9 (2.9–5.5)	4.0 (2.9–5.5)	4.2 (2.9–5.4)	0.897
Creatinine (μmol/L)	64 (58–81)	64 (56–83)	64 (59–79)	0.509
Albumin (g/L)	31 (26–36)	29 (26–32)	33 (27–37)	0.003
TBL (μmol/L)	26 (16–46)	35 (19–57)	22 (15–41)	0.011
ALT (U/L)	28 (20–44)	29 (20–41)	27 (20–47)	0.700
AST (U/L)	51 (34–75)	53 (34–77)	50 (35–68)	0.708
GGT (U/L)	134 (61–332)	124 (70–302)	140 (51–378)	0.967
SNA (mmol/L)	139 (137–141)	138 (136–140)	140 (138–142)	0.018
PT (s)	13.7 (12.5–15.5)	14.6 (13.2–16.4)	12.8 (12.2–14.6)	<0.001
PTA (%)	74 (60–87)	67 (56–79)	82 (67–91)	<0.001
INR	1.18 (1.07–1.34)	1.24 (1.12–1.36)	1.10 (1.03–1.26)	<0.001
PVD (mm)	13.9 (12.2–15.7)	14.1 (12.4–15.8)	13.8 (12.2–15.6)	0.563
SVD (mm)	8.4 (6.7–10.0)	8.5 (7.2–10.0)	8.3 (6.5–9.9)	0.267
SLD (mm)	124 (108–136)	130 (117–142)	117 (101–132)	<0.001
CTSV (cm^3^)	638 (406–839)	793 (589–1,007)	491 (344–688)	<0.001
CTLV (cm^3^)	1,239 (1,001–1,599)	1,231 (962–1,621)	1,263 (1,005–1,589)	0.727
SVER	2.86 (1.60–4.26)	3.70 (2.43–5.31)	2.11 (1.05–3.32)	<0.001
MELD score	11 (8–15)	13 (10–16)	9 (8–13)	<0.001
PSVR	171.6 (88.7–300.1)	120.9 (68.7–212.7)	230.5 (138.0–404.1)	<0.001
PSDR	843.6 (513.7–1,169.2)	699.3 (453.4–976.2)	943.2 (650.9–1,390.3)	<0.001
APRI	1.29 (0.76–2.45)	1.40 (0.91–2.64)	1.19 (0.72–2.06)	0.058
AAR	1.71 (1.32–2.36)	1.81 (1.38–2.46)	1.65 (1.25–2.32)	0.223

AAR, aspartate aminotransferase-to-alanine aminotransferase ratio; ALT, alanine aminotransferase; APRI, aspartate aminotransferase-to-platelets ratio index; AST, aspartate aminotransferase; CTLV, CT-measured liver volume; CTSV, CT-measured spleen volume; GGT, γ-glutamyl transpeptidase; HREV, high-risk esophageal varices; INR, prothrombin international normalized ratio; MELD, the model for end-stage liver disease; PLT, platelet; PSDR, platelet-to-spleen diameter ratio; PSVR, platelet-to-spleen volume ratio; PT, prothrombin time; PTA, prothrombin activity; PVD, portal vein diameter; RBC, red blood cell; SLD, spleen long diameter; SNA, serum sodium; SVD, splenic vein diameter; SVER, spleen volume enlargement ratio; TBL, total bilirubin; WBC, white blood cell.

### Construction of the nomogram model

**Figure 2:**
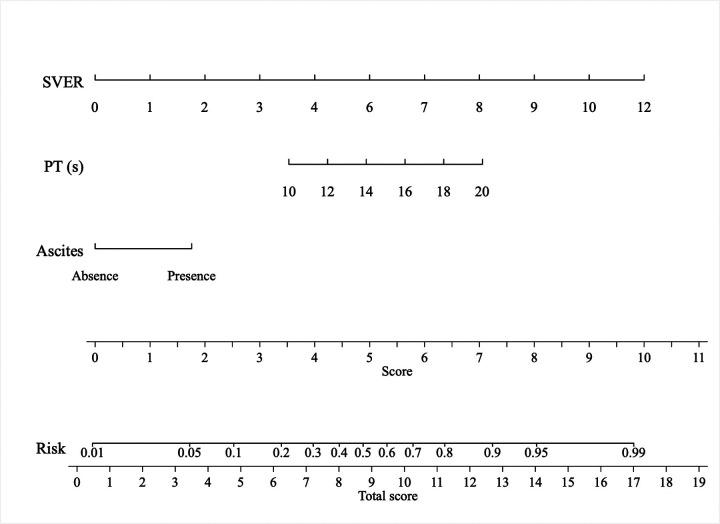
Nomogram for predicting high-risk esophageal varices in patients with liver cirrhosis. PT, prothrombin time; SVER, spleen volume enlargement rate.

As shown in Table 2, univariate logistic regression analysis revealed that 11 laboratory and imaging parameters were significantly associated with HREVs, including ascites (*β* = 1.274, *P* < 0.001), RBC count (*β* = −0.417, *P* = 0.092), PLT count (*β* = −0.010, *P* = 0.004), albumin (*β* = −0.078, *P* = 0.005), PT (*β* = 0.247, *P* = 0.002), PTA (*β* = −0.037, *P* < 0.001), INR (*β* = 2.990, *P* = 0.001), SLD (*β* = 0.027, *P* = 0.001), CTSV (*β* = 0.003, *P* < 0.001), SVER (*β* = 0.477, *P* < 0.001), and MELD score (*β* = 0.140, *P* = 0.001). Multivariate logistic regression analysis of these significant variables demonstrated that the presence of ascites [odds ratio (OR) = 2.656, 95% confidence interval (CI): 1.224–5.763], prothrombin time (OR = 1.217, 95% CI: 1.043–1.419), and SVER (OR = 1.589, 95% CI: 1.276–1.979) were independent predictors of HREVs (Table 3). Therefore, the probability of HREV was calculated using the following equation: ln [*P*/(1−*P*)] = 0.977 × Presence of ascites + 0.196 × PT (s) + 0.463 × SVER−4.856. These independent factors were incorporated into a nomogram for predicting HREVs in patients with liver cirrhosis (Figure 2).

**Table 2:** Univariate logistic regression analyses to identify predictors for HREVs.

Variable	*β*	OR	95% CI	*P* *
Male sex	0.361	1.435	0.736–2.799	0.289
Age	−0.024	0.976	0.945–1.009	0.151
BMI	0.048	1.049	0.963–1.143	0.271
Ascites	1.274	3.576	1.793–7.131	**<0** **.** **001**
WBC	−0.128	0.88	0.734–1.056	0.170
RBC	−0.417	0.659	0.405–1.071	**0** **.** **092**
Hemoglobin	−0.005	0.995	0.981–1.009	0.468
PLT	−0.010	0.990	0.983–0.997	**0** **.** **004**
Nitrogen	0.001	1.001	0.974–1.029	0.941
Creatinine	0.002	1.002	0.997–1.007	0.437
Albumin	−0.078	0.925	0.876–0.977	**0** **.** **005**
TBL	0.005	1.005	0.997–1.014	0.211
ALT	−0.009	0.991	0.979–1.004	0.185
AST	0.003	0.997	0.993–1.002	0.224
GGT	−0.0001	1	0.999–1.001	0.885
SNA	−0.066	0.936	0.858–1.021	0.136
PT	0.247	1.280	1.098–1.494	**0** **.** **002**
PTA	−0.037	0.964	0.944–0.983	**<0** **.** **001**
INR	2.99	19.88	3.33–118.6	**<0** **.** **001**
PVD	0.023	1.023	0.898–1.166	0.735
SVD	0.095	1.100	0.938–1.290	0.239
SLD	0.027	1.027	1.011–1.043	**0** **.** **001**
CTSV	0.003	1.003	1.002–1.004	**<0** **.** **001**
CTLV	−0.0001	1	0.999–1.001	0.769
SVER	0.477	1.612	1.306–1.990	**<0** **.** **001**
MELD score	0.140	1.150	1.056–1.251	**0** **.** **001**

ALT, alanine aminotransferase; AST, aspartate aminotransferase; CI, confidence interval; CTLV, CT-measured liver volume; CTSV, CT-measured spleen volume; GGT, γ-glutamyl transpeptidase; HREV, high-risk esophageal varices; INR, prothrombin international normalized ratio; MELD, the model for end-stage liver disease; OR, odds ratio; PLT, platelet; PT, prothrombin time; PTA, prothrombin activity; PVD, portal vein diameter; RBC, red blood cell; SLD, spleen long diameter; SNA, serum sodium; SVD, splenic vein diameter; SVER, spleen volume enlargement rate; TBL, total bilirubin; WBC, white blood cell.

* Variables with univariate *P*-value <0.10 were included in the multivariate analysis.

*P*-value <0.10 are shown in bold.

**Table 3:** Independent predictive factors identified by multivariate analysis.

Variable	*β*	OR	95% CI	*P*
Ascites	0.977	2.656	1.224–5.763	0.013
PT	0.196	1.217	1.043–1.419	0.012
SVER	0.463	1.589	1.276–1.979	<0.001
Constant	−4.856	0.008	0.007–0.082	<0.001

CI, confidence interval; OR, odds ratio; PT, prothrombin time; SVER, spleen volume enlargement rate.

### Performance and internal validation

**Figure 3:**
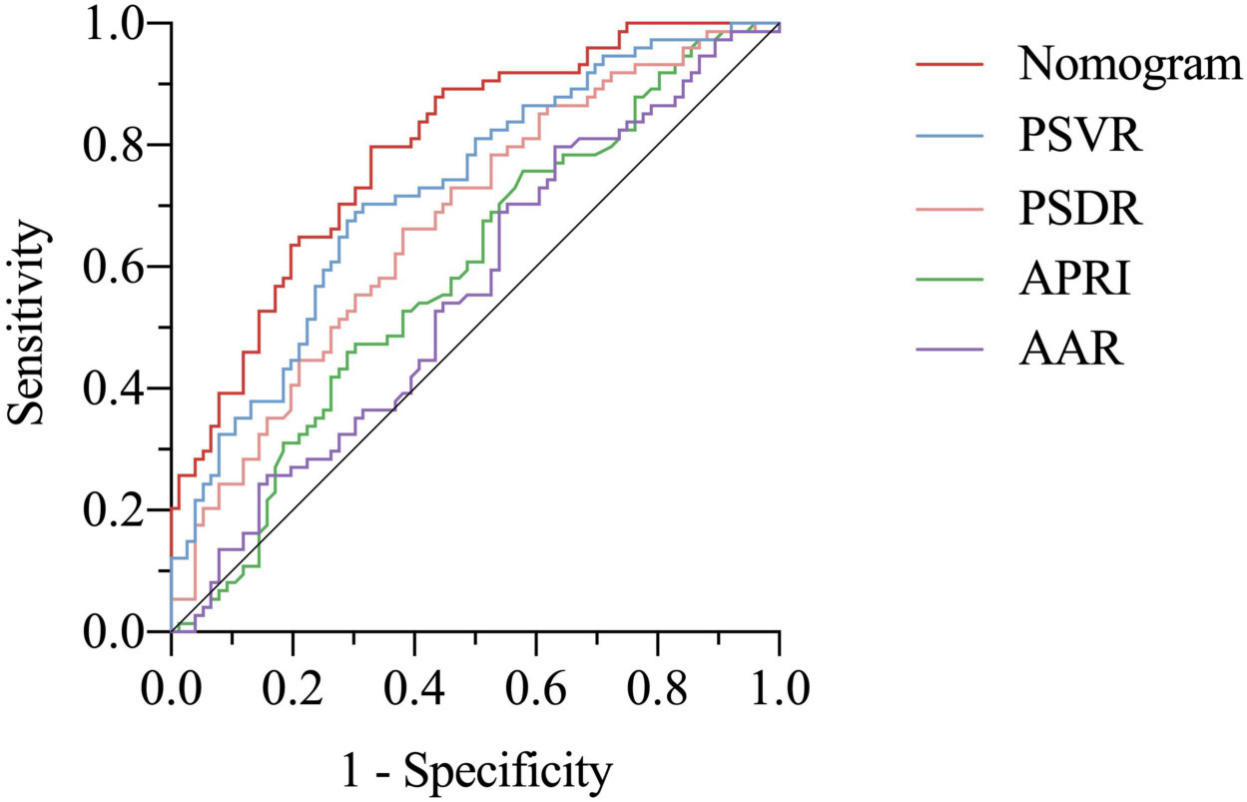
Receiver operating characteristic curves of the nomogram and other non-invasive models. AAR, aspartate aminotransferase-to-alanine aminotransferase ratio; APRI, aspartate aminotransferase-to-platelets ratio index; PSDR, platelet-to-spleen diameter ratio; PSVR, platelet-to-spleen volume ratio.

Figure 3 illustrates the ROC curves for the nomogram model and other non-invasive predictive models. The area under the ROC curve (AUC) for the nomogram model was 0.793 (95% CI: 0.723–0.863), indicating good discriminative ability. The optimal probability cutoff value was determined to be 0.431 by maximizing the Youden index. The nomogram model demonstrated an accuracy of 0.733, with a sensitivity of 0.797, specificity of 0.671, PPV of 0.702, and NPV of 0.773. The DeLong test indicated a significantly higher AUC for the nomogram model than that of PSVR (0.724, *P* = 0.061), PSDR (0.673, *P* = 0.019), APRI (0.590, *P* < 0.001), and AAR (0.558, *P* = 0.001). Additionally, the nomogram model exhibited superior accuracy, specificity, PPV, and NPV compared to these models (Table 4).

**Table 4:** Discriminative ability of the constructed model and other models.

Model	AUC (95% CI)	Sensitivity	Specificity	PPV	NPV	Accuracy	Cutoff	Comparison
Nomogram	0.793 (0.723–0.863)	0.797	0.671	0.702	0.773	0.733	>0.421	Ref.
PSVR	0.724 (0.643–0.804)	0.703	0.684	0.684	0.703	0.693	<172.9	*P* = 0.061
PSDR	0.673 (0.587–0.758)	0.662	0.618	0.633	0.662	0.640	<848.9	*P* = 0.007
APRI	0.590 (0.498–0.681)	0.757	0.408	0.554	0.633	0.587	>0.905	*P* < 0.001
AAR	0.558 (0.465–0.650)	0.797	0.368	0.551	0.651	0.580	>1.345	*P* < 0.001

AUC, area under the curve; CI, confidence interval; NPV, negative predictive value; PPV, positive predictive value.

**Figure 4:**
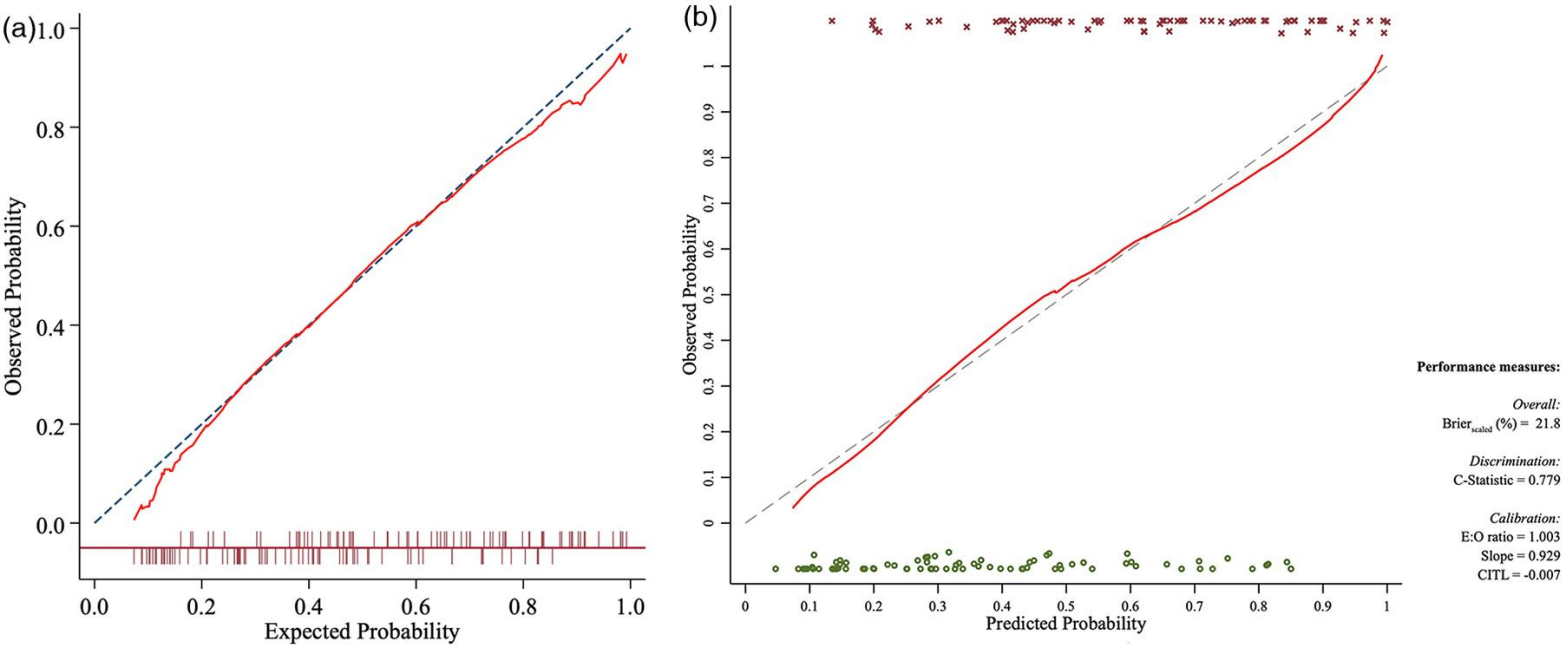
Calibration curves of the monogram model in the developing cohort **(a)** and bootstrapping internal validation **(b)**.

The calibration plot and Hosmer–Lemeshow test demonstrated no significant difference between the observed probabilities and those predicted by the nomogram model (Figure 4a, *P* = 0.247). Internal validation of the constructed nomogram model was performed using bootstrapping with 1,000 resampling iterations (Figure 4b), yielding a C-index (equivalent to the AUC) of 0.779 (95% CI: 0.714–0.853).

### Clinical utility

**Figure 5:**
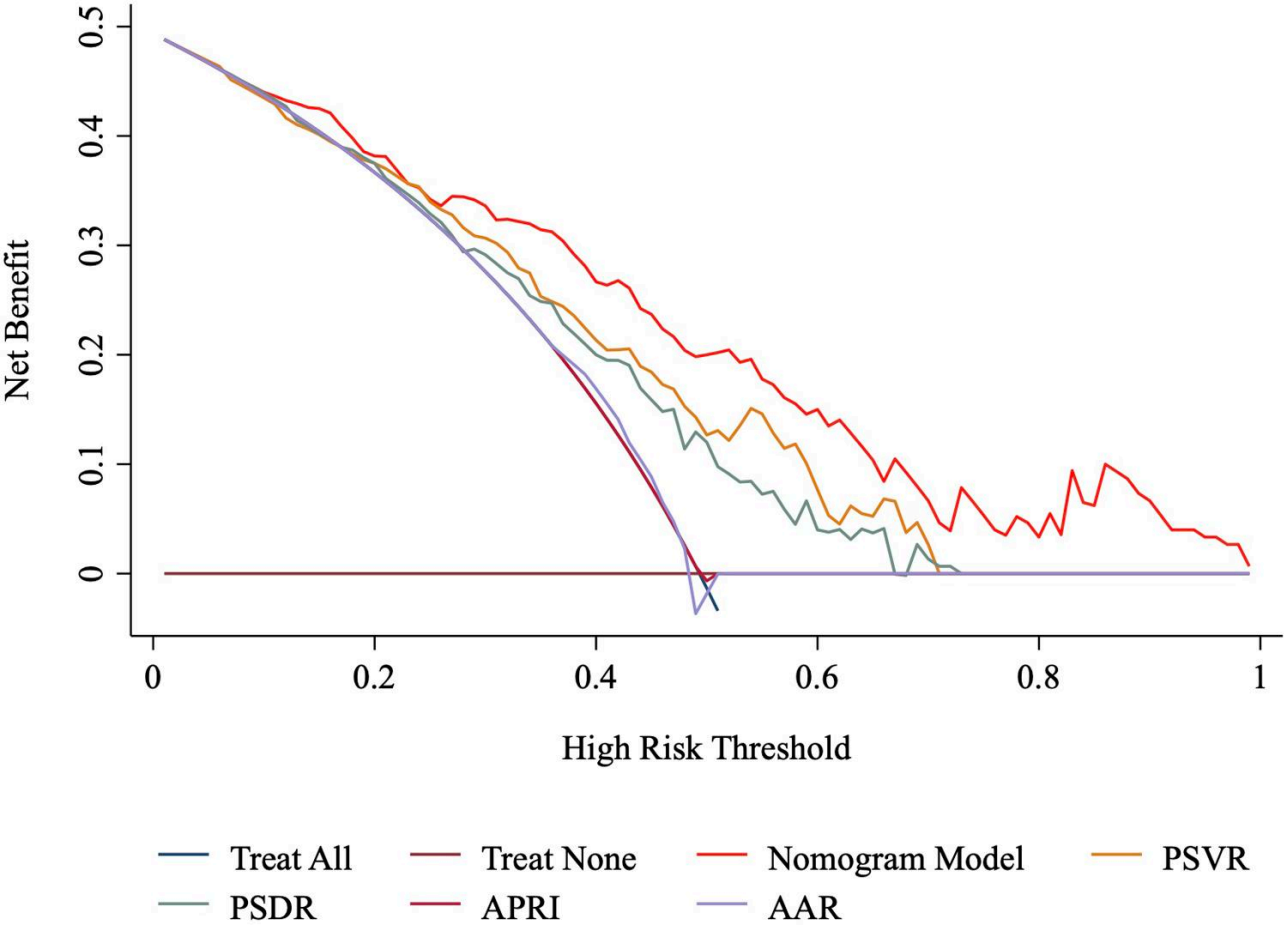
Decision curve analysis of the nomogram and other non-invasive models. AAR, aspartate aminotransferase-to-alanine aminotransferase ratio; APRI, aspartate aminotransferase-to-platelets ratio index; PSDR, platelet-to-spleen diameter ratio; PSVR, platelet-to-spleen volume ratio.

DCA was performed to evaluate the clinical utility of the nomogram model in predicting HREVs in patients with liver cirrhosis (Figure 5). The nomogram model demonstrated a greater net clinical benefit than other non-invasive models across a wide range of high-risk thresholds.

## Discussion

This study developed a novel nomogram model incorporating laboratory and imaging examinations to predict HREVs in patients with liver cirrhosis. The model demonstrated good discriminatory performance, calibration ability, and clinical utility, outperforming previously established indices such as the PSDR, PSVR, APRI, and AAR. The user-friendly nomogram may facilitate individualized HREV prediction, enabling timely evaluations and preventive treatments while potentially minimizing unnecessary upper gastrointestinal endoscopy.

The nomogram model included three independent predictors of HREVs identified through multivariate analysis: ascites, PT, and SVER. Ascites, a common manifestation of decompensated cirrhosis, was found in 74.3% of HREV patients, compared to 44.7% in non-HREV patients. Previous studies have recognized ascites as an independent predictive factor of EV bleeding in viral hepatitis-related cirrhosis (20), which aligns with our findings. Accordingly, ascites has been incorporated into nomogram models for predicting the risk of EV bleeding (21, 22). PT, another independent predictor, was significantly prolonged in HREV patients, likely due to reduced hepatic synthesis of coagulation factors associated with the progression of cirrhosis (23). Previous research has also indicated a positive correlation between prolonged PT and EV bleeding, further supporting its predictive ability when combined with other parameters (21, 24–26).

Spleen volume enlargement, which develops as a consequence of portal hypertension and collateral circulation, indirectly reflects portal pressure and is closely linked to the severity of EV and the risk of bleeding (27, 28). Our study employed the SVER, a novel parameter that provides a more accurate reflection of spleen enlargement than clinical assessment of palpable splenomegaly. SVER has been recently proposed and investigated for its predictive value in EVs (29), EV bleeding (25), and HREVs (30). Consistent with the findings of Yang *et al*. (30), our study revealed significantly higher SVER values in HREV patients compared to non-HREV patients. Previous models and the current nomogram incorporating SVER and additional parameters have demonstrated good to excellent diagnostic performance, underscoring the feasibility and clinical relevance of SVER-based predictive models.

In addition to SVER, several indices combining splenic parameters with platelet counts, such as PSDR and PSVR, have been shown to correlate with EV severity and serve as effective non-invasive predictors of both EVs and HREVs (10, 31–33). A meta-analysis by Chen et al. evaluated the diagnostic performance of PSDR in patients with liver cirrhosis, finding an area under the summary receiver operating characteristic curve (AUSROC) of 0.8132, with pooled sensitivity and specificity of 0.78 and 0.68, respectively (34). Another meta-analysis indicated that PSDR outperformed platelet count and spleen length alone in diagnosing varices at high risk of bleeding (35). Lee et al. developed the spleen volume-to-platelet ratio index in HBV-related compensated cirrhosis, achieving a sensitivity of 69.4% and a specificity of 78.5% for detecting high-risk varices at a cutoff value >1.63 (14). Long-term follow-up further revealed a cumulative 5-year incidence of variceal bleeding of 12% among patients categorized as high risk by this index (14). Yu et al. reported that PSVR performed superiorly compared to PSDR, spleen volume, spleen diameter, and platelet count in predicting EVs and HREVs among patients with hepatitis B liver cirrhosis (10). In our study, the nomogram model was compared to PSVR, PSDR, and commonly used indices such as APRI and AAR (19). The nomogram model showed higher AUC, sensitivity, PPV, NPV, and accuracy than these other models, indicating superior discriminative ability for predicting HREVs. Decision curve analysis further revealed that the nomogram model provided greater net clinical benefit than alternative models, suggesting enhanced clinical efficacy.

In addition to spleen volume, various models incorporating stiffness measurements have been developed to identify patients at low risk of HREVs and thereby reduce unnecessary endoscopy examinations, which are invasive, costly, and uncomfortable. The Baveno VI consensus suggests that endoscopy can be avoided in patients with compensated advanced chronic liver disease (cACLD) who have a liver stiffness measurement (LSM) <20 kPa and a platelet count >150,000/μl, as these patients are less likely to have HREVs (5). The diagnostic performance of the Baveno VI criteria has been validated across diverse populations (36, 37). A clinical trial further demonstrated that combining the Baveno VI criteria with spleen stiffness measurement (SSM) ≤46 kPa effectively ruled out HREVs (38). Recently, the Baveno VII consensus recommended an SSM threshold of ≤40 kPa to safely rule out HREVs and avoid endoscopic screening (39). This updated algorithm outperformed other non-invasive models, including the Baveno VI criteria, liver stiffness measurement-longitudinal spleen diameter-to-platelet ratio score (LSPS), and PSDR, by sparing more unnecessary endoscopy while reducing the misclassification of HREVs (40). Despite their excellent performance, liver and spleen stiffness measurements obtained via transient elastography may be unsuccessful due to factors such as obesity, ascites, and narrow intercostal spaces, yielding uninterpretable measurements in nearly 20% of cases (41). Newer modalities like point shear wave elastography (pSWE) and 2D shear wave elastography (2D-SWE) are less affected by these factors and have shown accurate predictions of HREVs (42, 43). However, these methods are not yet widely accessible, particularly in less developed regions. In contrast, the indicators in our model can be easily obtained through physical examination (ascites), laboratory testing (PT), and commonly performed imaging studies (SVER). Therefore, our nomogram model offers an effective, non-invasive, and easily assessable tool for identifying HREVs in patients with liver cirrhosis.

Several limitations of our study should be acknowledged. First, its single-center design may have introduced selection bias. Second, the relatively small sample size limited our ability to validate the nomogram model in an independent cohort. Finally, while we developed the model within a population with diverse etiologies of liver cirrhosis, its predictive performance in cirrhosis arising from specific etiologies, such as viral hepatitis (the leading cause in China), requires further confirmation.

## Conclusions

We developed a nomogram model based on ascites, PT, and SVER, which demonstrated good predictive performance for HREVs in patients with liver cirrhosis. This nomogram provides an effective, non-invasive, and user-friendly tool for diagnosing HREVs, thereby facilitating preventive treatment of variceal bleeding and reducing unnecessary endoscopic examinations.

## Data Availability

The raw data supporting the conclusions of this article will be made available by the authors, without undue reservation.
